# Lower product of magnesium × potassium is associated with higher mortality in chronic hemodialysis patients: a cohort study

**DOI:** 10.1038/s41598-023-49372-y

**Published:** 2023-12-13

**Authors:** Jui-Yi Chen, Ming-Yan Jiang, Yun-Ting Huang, Jyh-Chang Hwang

**Affiliations:** 1 Division of Nephrology, Department of Internal Medicine, Chi Mei Medical Center, No. 901, Zhonghua Rd., Yongkang Dist., Tainan City, Taiwan; 2 Department of Health and Nutrition, Chia Nan University of Pharmacy and Science, Tainan, Taiwan; 3 Department of Hospital and Health Care Administration, Chia Nan University of Pharmacy and Science, Tainan, Taiwan

**Keywords:** Medical research, Nephrology

## Abstract

This study aimed to investigate the Mg × K product on the mortality risk of hemodialysis patients with concomitant hypokalemia and lower magnesium levels. This was a prospective observational study of patients in a HD center in southern Taiwan. A total of 444 HD patients were divided into 5 groups by the Mg × K product: group 1, bottom quintile, median Mg × K: 7.87, IQR: 7.03–8.12 (n = 89, age: 64 ± 13 years old); group 2, median Mg × K: 9.37, IQR: 8.97–9.86 (n = 89, age:62 ± 13 years old); group 3, median Mg × K: 10.95, IQR: 10.50–11.26 (n = 89, age:64 ± 13 years old); group 4, median Mg × K: 12.30, IQR: 11.87–12.82 (n = 89, 61 ± 12 years old); and group 5, top quintile, median Mg × K: 14.92, IQR:14.07–16.23 (n = 88, 62 ± 11 years old). The patients were followed up for 2 years to determine the risk of all-cause mortality. Patients with a lower Mg × K product had more comorbidities, malnutrition-inflammation status, and a higher mortality risk. Using multivariable Cox regression analysis, a higher Mg × K [HR, 0.89; 95%CI (0.81–0.98)] was found to be an independent predictor of better survival. HD patients with a lower Mg × K product had more comorbidities, a marked malnutrition-inflammation status, and were associated with long-term mortality. A higher Mg × K value is a favorable survival factor.

## Introduction

For patients with chronic kidney disease (CKD), there exists a U-shaped curve, highlighting increased mortality rates with blood potassium levels below 4.0 mEq/L and above 4.5 mEq/L^1^. Conversely, hypokalemia in hemodialysis (HD) patients is associated with worse outcomes, including mortality and cardiac events^2^. A low pre-dialysis serum potassium (K) level, relative to optimal ranges, poses a significant risk factor for mortality^2^, and post-dialysis hypokalemia increases the incidence of sudden cardiac arrest and cardiac death^3^. Notably, in HD patients, all-cause mortality risk escalates with rising serum potassium levels, becoming significant at potassium levels exceeding 5.7 mEq/l^4^.

On the other hands, hospitalized patients exhibiting a magnesium (Mg) level below 1.7 mg/dL face two pronounced risks independently: a heightened probability of in-hospital mortality and an extended hospital stay. Moreover, hypermagnesemia may compromise cardiac function, specifically impinging on both the systolic contraction and diastolic relaxation phases^5^. A high Mg level, reaching at 2.3 mg/dL or higher, also correlates directly with mortality risk^6^. There's also a noted correlation between hypermagnesemia detected upon emergency department admission and early in-hospital mortality^7^.

In contrast, the ionized fraction of magnesium (Mg) in dialysis patients diminishes due to elevated circulating levels of phosphate, citrate, and sulfate which form complexes with Mg^8^. End-stage renal disease (ESRD) patients with hypomagnesaemia may encounter disruptions in energy homeostasis and lipid/glucose metabolism^9^, potentially correlating with ischemic heart disease^10^. In addition, dialysis patients suffering from hypomagnesaemia tend to face poor outcomes^11^.

Drawing from the clinical and experimental studies, the interrelationships between Mg and K in cardiac tissue holds substantial clinical relevance, particularly concerning arrhythmias, and myocardial infarction^12^. However, the clinical implications of simultaneous dyskalemia and dysmagnesaemia in chronic HD patients are yet to be fully understood. An imbalance in serum K and Mg levels is common among HD patients. Besides, serum magnesium and potassium levels are positively correlated, which are crucial for bodily functions^12^. Despite this, the collective impact of pre-HD serum Mg and K product on survival rates in patients undergoing maintenance HD has not been explored to date. Given the easy accessibility of these two markers in clinical practice, our study aims to stratify HD patients by the product of Mg × K and examine the long-term prognostic implications of varying Mg × K levels in this population.

## Results

### Characteristics of the study participants

A total of 478 consecutive patients were eligible for this study, and after applying the exclusion criteria 444 individuals were enrolled. The mean patient age was 62 years. A positive linear correlation was noted between serum K and Mg levels (beta = 0.29, 95% confidence interval [0.38, 0.73], *p* < 0.001), and the average serum Mg levels were 2.5 mg/dL. Additionally, the total mortality rate was 14.9% (n = 66). HD patients were divided into quintiles based on the different Mg × K products, which are listed in Table 1. For these five groups, shorter HD vintage and lower normalized protein catabolism rate (nPCR) were noted in group 1 compared to the other four groups. We also found that group 1 had higher high sensitity-C reactive protein (hs-CRP) levels, more comorbidities, and lower albumin levels, whereas groups 4 and 5 had higher albumin and prealbumin levels (Table 1). Group 1 was also characterized by the lowest levels of phosphate, blood urea nitrogen (BUN), creatinine, and Mg among the five groups. The baseline serum magnesium and potassium levels within the normal range for each group are also detailed in Table 1. Further, using multivariate logistic regression tests, group 1 showed a positive relationship with shortened HD vintage, lower nPCR, serum creatinine, and higher hs-CRP (Table 2).

**Table 1:** Basic demographic characteristics of the five groups by different Mg × K production for dialysis patients.

Variables	Group 1 (n = 89)	Group 2 (n = 89)	Group 3 (n = 89)	Group 4 (n = 89)	Group 5 (n = 88)
Demographic factors					
DM (%)	51	42	47	46	44
Male (%)	61	62	55	52	52
Age at study (years)	64 ± 13	62 ± 13	64 ± 13	61 ± 12	62 ± 11
HD vintage, (months)	4.7 ± 5.4	7.1 ± 6.1	7.3 ± 5.8	6.4 ± 5.7	7.1 ± 5.5
nPCR* (g/kg/day)	1.29 ± 0.30	1.37 ± 0.28	1.37 ± 0.26	1.44 ± 0.31	1.45 ± 0.27
SBP (mmHg)	134.5 ± 27.5	137.7 ± 33.4	134.8 ± 28.8	137.9 ± 27.7	137.1 ± 24.1
DBP (mmHg)	73.6 ± 12.8	73.7 ± 15.4	72.9 ± 14.0	75.6 ± 16.9	75.0 ± 13.5
UF (L/session)	2.4 ± 1.0	2.3 ± 1.0	2.4 ± 0.9	2.4 ± 1.0	2.3 ± 0.9
BMI (kg/m^2^)	23.0 ± 3.5	23.8 ± 4.7	23.3 ± 3.9	23.1 ± 4.6	22.2 ± 3.6
Clinical comorbidity (%)					
CAD (%)	40	34	28	24	30
CHF (%)	12	9	9	6	8
Peripheral vascular disease	13	8	4	7	6
Liver cirrhosis and/or hepatoma	3	3	6	6	1
Davies comorbidity score (%)					
0	26	33	26	31	39
1–2	49	51	57	55	44
> = 3	25	17	17	13	17
Laboratory data					
hs-CRP (mg/L)	6.3 (2.1–12.6)	4.0 (2.0–6.3)	3.8 (1.4–10.5)	4.3 (1.4–10.9)	2.1 (1.0–5.3)
Median (1st–3rd QR)					
Pre-albumin (mg/dl)	28.5 ± 8.7	26.7 ± 7.1	30.3 ± 8.1	31.8 ± 7.6	32.9 ± 6.3
Albumin (g/dl)	3.64 ± 0.50	3.88 ± 0.30	3.86 ± 0.29	4.00 ± 0.33	4.00 ± 0.28
Product of Mg × K	7.87(7.03–8.12)	9.37(8.97–9.86)	10.95*(10.50–11.26)	12.3(11.87–12.82)	14.92(14.07–16.23)
iPTH, median, pg/mL	243.6	234.7	371.5	273.7	327.0
(1st–3rd QR)	(114.3–652.2)	(95.6–458.9)	(138.0–655.1)	(113.0–604.0)	(177.5–758.4)
Sodium (mmol/dL)	138.6 ± 4.5	139.1 ± 2.7	139.4 ± 3.0	138.8 ± 2.7	139.0 ± 2.8
Potassium (mmol/dL)	3.5 ± 0.5	4.0 ± 0.4	4.4 ± 0.4	4.7 ± 0.5	5.2 ± 0.6
Magnesium (mg/dl)	2.1 ± 0.3	2.4 ± 0.2	2.5 ± 0.2	2.7 ± 0.3	3.0 ± 0.4
Corrected Calcium (mg/dl)^#^	9.1 ± 0.8	9.3 ± 0.8	9.3 ± 0.7	9.4 ± 0.9	9.3 ± 0.8
Phosphate (mg/dl)	4.3 ± 1.3	4.9 ± 1.3	5.0 ± 1.1	5.2 ± 1.5	5.4 ± 1.4
Cholesterol (mg/dl)	163 ± 43	166 ± 40	160 ± 39	174 ± 46	174 ± 43
BUN (mg/dl)	57.3 ± 17.3	60.3 ± 15.4	67.0 ± 15.6	68.5 ± 18.0	70.7 ± 18.4
Creatinine (mg/dl)	9.0 ± 3.2	10.4 ± 2.4	10.5 ± 2.8	10.9 ± 2.8	11.0 ± 2.8
Uric acid (mg/dl)	6.7 ± 1.9	6.9 ± 1.7	7.0 ± 1.5	7.1 ± 1.6	7.1 ± 1.4
Hemoglobin (g/L)	9.8 ± 1.6	10.2 ± 1.4	10.1 ± 1.5	10.2 ± 1.2	10.4 ± 1.6
K: 3.5–4.0 (mmol/dL) (n) (%)	7 (7.9)	11 (12.4)	4 (4.5)	12 (13.5)	10 (11.4)
Mg: 2.1–2.4 (mg/dl) (n) (%)	31 (34.8)	36 (40.4)	29 (32.6)	31(34.8)	24 (27.3)
Medications, n (%)					
Mg containing agents	9 (10)	4 (4)	13 (15)	11 (12)	8 (9)
Diuretics	41 (46)	42 (47)	43 (48)	35 (39)	30 (34)
Proton pumping agents	12 (13)	4 (4)	12 (13)	6 (7)	8 (9)
Vitamin D3	28 (32)	30 (34)	38 (43)	37 (42)	37 (42)

BMI, Body mass index; BUN, Blood urea nitrogen; CAD, Coronary artery disease; CHF, Congestive heart failure; DBP, Diastolic blood pressure; DM, Diabetes Mellitus; HD, Hemodialysis; hs-CRP, High sensitivity C-reactive protein; iPTH, Intact parathyroid hormone nPCR, Normalized protein catabolism rate; SBP, Systolic blood pressure; UF, Ultrafiltration.

^#^Corrected Ca = [0.8 x (normal albumin − patient's albumin)] + serum Ca level.

**Table 2:** Multivariate logistic regression tests for evaluation of the characteristics of Group 1.

	Univariate				Multivariate			
	95% CI				95% CI			
	OR	Lower	Higher	*p*	OR	Lower	Higher	*p*
HD vintage, year	0.922	0.878	0.969	0.001	0.931	0.881	0.984	0.011
nPCR, g/kg/day	0.213	0.082	0.552	0.001	0.28	0.107	0.738	0.01
Hs-CRP, mg/dL	1.012	1.003	1.022	0.01	1.014	1.001	1.026	0.029
Creatinine, mg/dL	0.793	0.72	0.873	< 0.001	0.834	0.749	0.928	0.001
Diabetes mellitus, DM = 1	1.278	0.802	2.037	0.301	0.681	0.388	1.193	0.179
Albumin, g/dL	0.119	0.058	0.245	< 0.001				
Prealbumin, mg/dL	0.955	0.925	0.986	0.005				
Intact PTH, pg/mL	1	0.999	1	0.562				
Sex, male = 1	1.263	0.786	2.03	0.335				
BUN, mg/dL	0.967	0.952	0.982	< 0.001				
Calcium, mg/dL	0.481	0.349	0.663	< 0.001				
Phosphate, mg/dL	0.589	0.48	0.722	< 0.001				
Hemoglobin, g/dL	0.829	0.704	0.975	0.024				

HD, Hemodialysis; nPCR, Normalized protein catabolism rate; CRP, C-reactive protein; intact PTH, Intact parathyroid hormone; DM, Diabetes mellitus, BUN, Blood urea nitrogen.

### Associated factors related to the product of Mg × K

The Mg × K product showed a positive correlation with nPCR, serum albumin, BUN, phosphate, creatinine, and prealbumin, but a negative correlation with hs-CRP. After adjusting for age, sex, and HD vintage, Mg × K still showed a positive correlation with nPCR and a negative correlation with hs-CRP (Table 2). An OR of 0.28 might imply that a lower nPCR is associated with certain characteristics of Group 1.

### Survival comparisons among five groups

**Figure 1:**
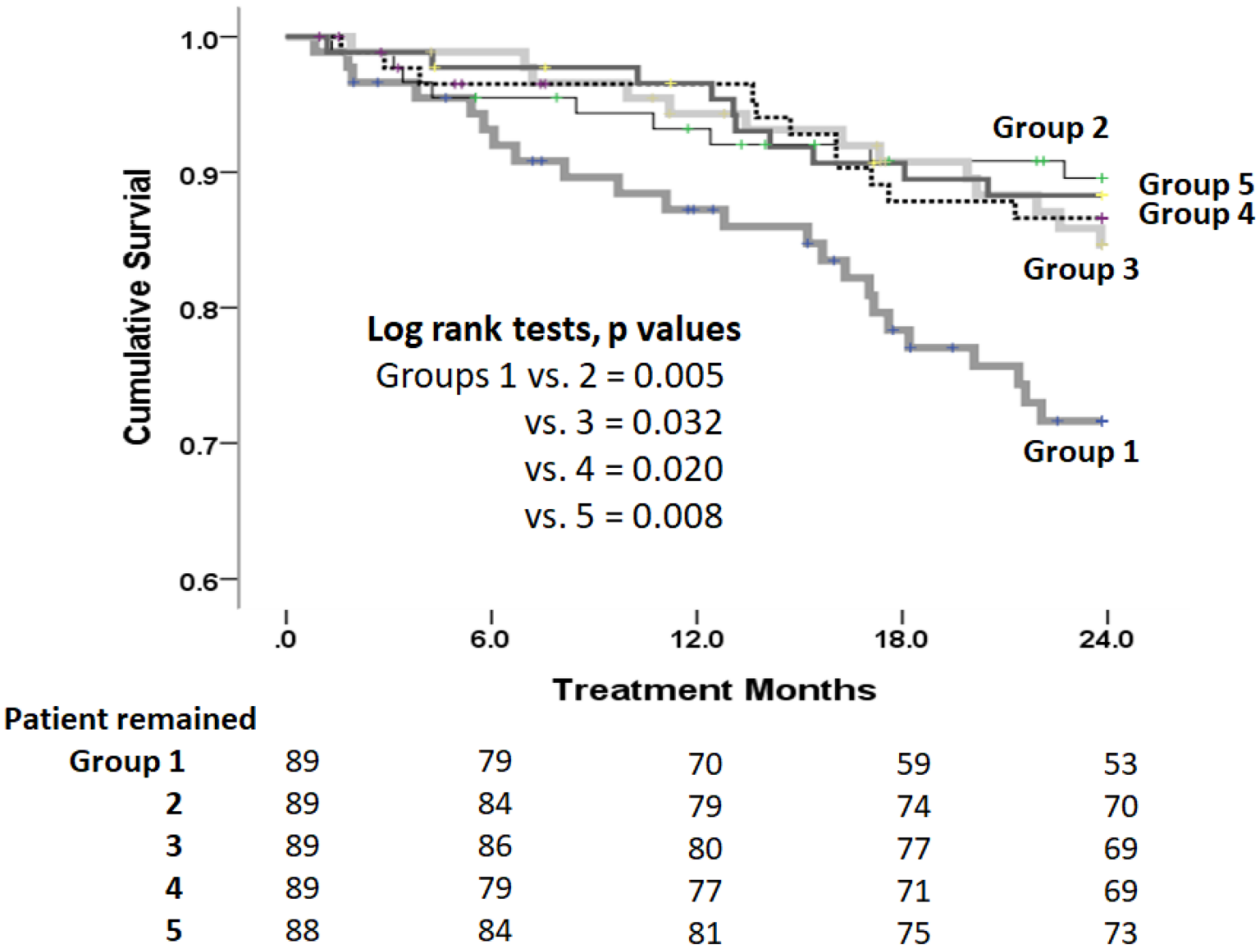
Kaplan–Meier survival analysis of cumulative all-cause mortality among five groups of patients based on level of Mg × K product. After 2 years’ follow-up, group 1 showed a lower cumulative survival rate than the other four groups. Except for group 1, all the other four groups did not differ from one another in cumulative survival.

Figure 1 shows the survival curves for the five Mg × K groups. After a median follow up of 20.91 months, group 1 showed worse survival compared to the other four groups (Fig. 1). In addition, in univariate Cox proportional model analysis, a lower Mg × K product, old age, lower prealbumin level, hypoalbuminemia, and high hs-CRP were significant factors associated with increased mortality risk, whereas in multivariate Cox proportional analysis adjusted for potential confounding factors, low Mg × K was still an independent risk factor for mortality (Table 3, models 1 and 2), but it lost power of predictor for mortality after including serum albumin and hs-CRP into analysis (model 3).

**Table 3:** Multivariate Cox Proportional hazard method for evaluation the risk factor for predicting mortality.

	Multivariate (model 1)				Multivariate (model 2)				Multivariate (model 3)			
	HR	95% CI		*p*	HR	95% CI		*p*	HR	95% CI		*p*
		lower	upper			lower	upper			lower	upper	
Mg x K*	0.89	0.812	0.976	0.013	0.88	0.806	0.961	0.004	0.95	0.865	1.043	0.282
Age, year	1.038	1.015	1.062	< 0.001	1.029	1.005	1.054	0.017	1.039	1.014	1.064	0.002
Diabetes mellitus, y or n	1.568	0.925	2.658	0.095	1.335	0.792	2.249	0.279	1.531	0.883	2.653	0.129
Sex, male = 1	0.957	0.584	1.567	0.86	0.927	0.567	1.516	0.763	1.031	0.609	1.744	0.911
Hemodialysis vintage, year	0.995	0.949	1.044	0.848	0.99	0.944	1.038	0.664	0.985	0.935	1.038	0.57
davies comorbidity score					1.285	1.143	1.445	< 0.001				
Serum albumin, g/dL									0.407	0.249	0.666	< 0.001
hs-CRP, mg/L									1.015	1.009	1.022	< 0.001
nPCR, g/kg/day												
Serum prealbumin, mg/dL												

	Univariate				Multivariate			*p*				
	HR	95% CI		*p*	HR	95% CI						
		lower	upper			lower	upper					
Mg x K	0.886	0.807	0.972	0.011	0.89	0.812	0.976	0.013				
Age, year	1.041	1.019	1.064	< 0.001	1.038	1.015	1.062	< 0.001				
Diabetes mellitus, y or n	1.792	1.097	2.928	0.02	1.568	0.925	2.658	0.095				
Sex, male = 1	0.903	0.557	1.464	0.678	0.957	0.584	1.567	0.86				
Hemodialysis vintage, year	0.975	0.932	1.021	0.281	0.995	0.949	1.044	0.848				
nPCR, g/kg/day	0.797	0.289	2.201	0.661								
Serum prealbumin, mg/dL	0.939	0.907	0.973	< 0.001								
Serum albumin, g/dL	0.286	0.2	0.408	< 0.001								
hs-CRP, mg/L	1.017	1.012	1.022	< 0.001								

Mg, Magnesium; K, Potassium; nPCR, Normalized protein catabolism rate; hs-CRP, high sensitity-C reactive protein. *, Mg × K(t0).

### Trend and consistency of the Mg × K product over the following 2 years

**Figure 2:**
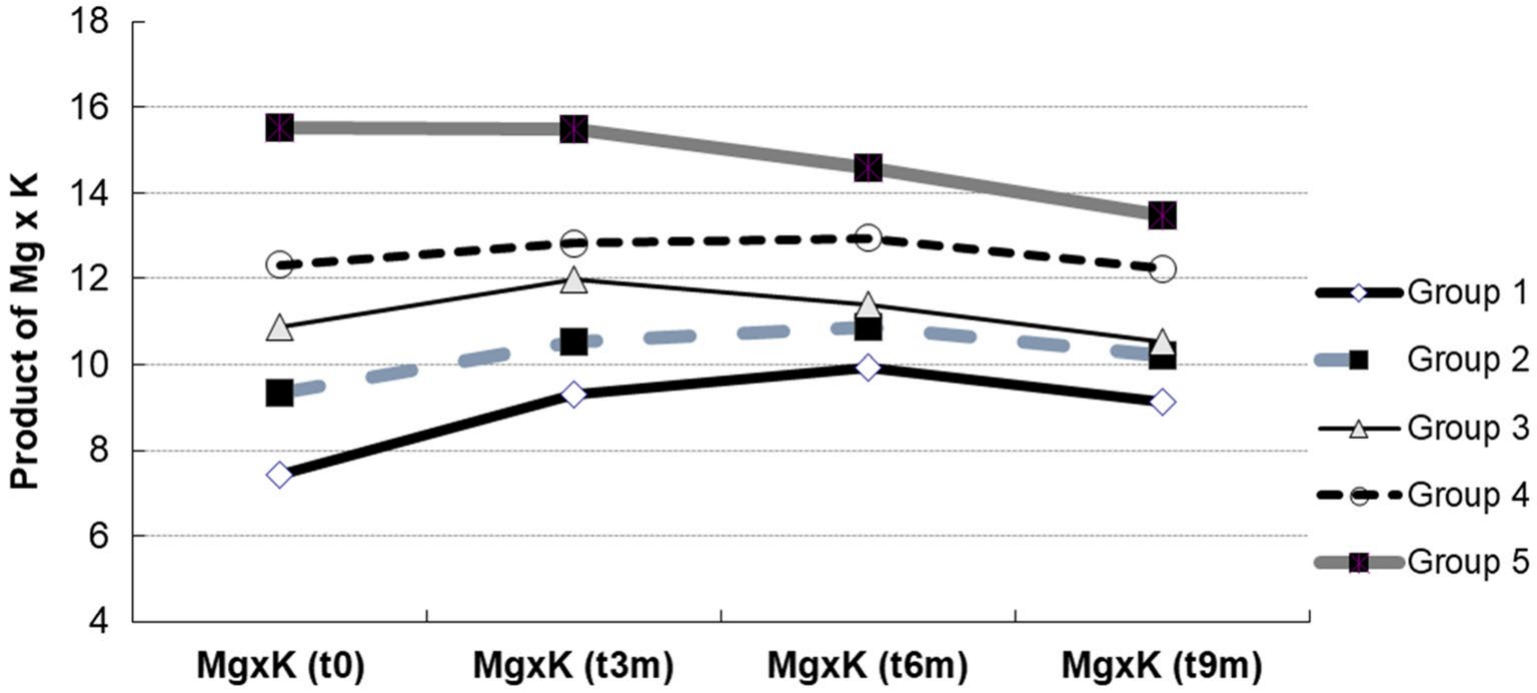
Trend and consistency of Mg × K products in following 1 years. Serum [Mg] and [K] were checked every 3 months. The products of Mg × K in five groups showed a consistent trend along this 9-month observation. Group 1 persistently had lower level than that of the other 4 groups.

In this study we checked serum Mg and K every 3 months. Figure 2 shows the trend and consistency of the Mg × K product in the five groups during this 9-month observation period. Group 1 persistently had the lower Mg × K product compared to the other four groups.

### Area under curve (AUC) of Mg × K is larger than the other two markers

**Figure 3:**
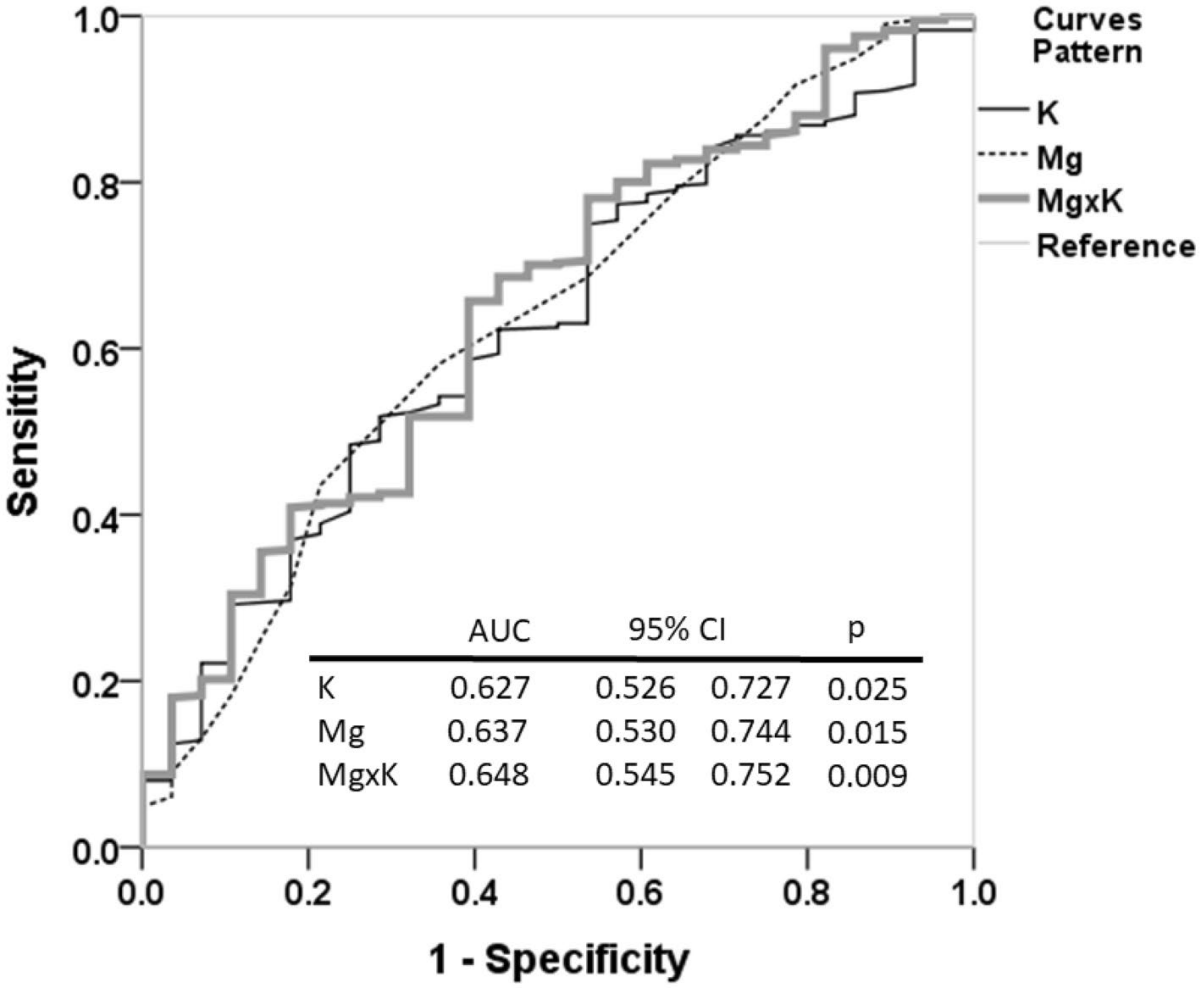
Comparison of Area Under the Curve for K, Mg, and Mg × K in Predicting Survival. When assessing the prognostic efficacy for survival using the AUC values, Mg × K demonstrated a higher AUC (Area Under the Curve) relative to either K or Mg alone, indicating its superior predictive capability.

By using receiver operating characteristic (ROC) curve for predicting survival probability, we observed that AUC value (0.648) of Mg × K was larger than either Mg (0.637) or K (0.627) (Fig. 3).

## Discussion

In this study we demonstrated a positive correlation between serum Mg and K in HD patients. These two biomarkers showed a parallel trend in chronic HD patients; patients with lower Mg would concomitantly have lower K, whereas those with higher Mg were also associated with higher K. ESRD patients with lower Mg × K product were characterized by shorter HD vintage, hypoalbuminemia, hypoprealbuminemia, hypophosphatemia, and lower creatinine levels, but by higher hs-CRP levels. Group 1 patients with the lowest Mg × K product were noted to have a higher mortality risk than other groups with higher Mg × K. We suggest that Mg × K is a good outcome predictor in chronic HD patients, with mortality risk being reduced 19% for every 1-unit increment in the Mg × K product. Thus, we hypothesized HD patients with lower Mg × K is equivalent to hypoalbuminemia and high CRP level, i.e., MIA syndrome, which make “Mg × K” lost of significance in predicting power in the analysis of Cox proportional method.

Since both Mg and K are excreted mainly through the kidneys, increased levels of these two electrolytes are more frequently encountered in patients with deteriorated glomerular filtration rate due to diminishing excretion. Compared to normal and supernormal counterparts, subnormal levels of these two cations are relatively rare in dialysis patients. Still, however, hypomagnesemia^13,14^ and hypokalemia^15^ have been reported in 6–39% and 11% of chronic HD patients, respectively. In our study, hypomagnesaemia (< 1.9 mg/dL) accounted for 3.1% of our chronic HD patients, while hypokalemia (< 3.5 mEq/dL) for 12.3%.

Hypomagnesaemia results primarily from gastrointestinal or urinary loss, although malnutrition and decreased nutritional intake may be another important mechanism. Dietary restriction in HD patients limits Mg intake, since higher K diet contains more Mg. Poor appetite and reduced food intake in some dialysis patients reduce Mg absorption. Proton pump inhibitors (PPI) have also been reported to inhibit intestinal absorption of Mg by disrupting active transport by transient receptor potential melastatin-6 and -7 channels^16^, although this could not explain why hypomagnesaemia was featured in group 1 patients as only a few of these patients were prescribed with PPI. Hypomagnesaemia may also result from inflammation and malnutrition in HD^17,18^ and peritoneal dialysis patients^19^. Lower serum albumin and higher hs-CRP levels in group 1 patients were evidence of ongoing subclinical inflammation and/or infection status. Hypomagnesaemic dialysis patients had a strong association with mortality risk^20^, especially those with hypoalbuminemia, which infers the link between hypomagnesaemia and the severity of underlying comorbidities.

Several mechanisms have been proposed to explain the association between lower serum Mg and higher mortality in HD patients, especially in those with hypoalbuminemia^17^. Mg deficiency, known to prompt endothelial dysfunction, vascular calcification and atherosclerosis, plays a critical role in cardiovascular pathology^21,22^. By fostering an inflammatory, pro-thrombotic, and pro-atherogenic environment, low magnesium levels inhibit endothelial proliferation, thereby potentially contributing to the onset and progression of cardiovascular disease^23^. (Supplement Table 2).

Mg inadequacy has been reported to lead to inflammation and to be related to insulin resistance and metabolic syndrome^24^. Deficient tyrosine kinase activity within insulin receptors leads to impaired regulation of both insulin-mediated glucose uptake and vascular tone modulation^25^.

Hypokalemia may also be caused by poor intake or over-excretion through stool or urine. Among the five Mg × K groups, the percentage of diuretics prescription and oliguric states were similar; therefore, urine loss was not a prominent factor for hypokalemia in these HD patients. Since all other counterparts with higher K also received the same dialysate during the HD sessions with a 2.0 mEq/L concentration of K, excess flux through the HD process may not have a role in lowering K in group 1 patients. In addition, no diarrhea or vomiting were observed during the study period. Therefore, it is logical to hypothesize that hypokalemia in group 1 may have resulted from a lack of nourishment and various degrees of inflammation^15^. A lower K has been suggested as a surrogate marker of malnutrition and is related to mortality^15^. For patients undergoing dialysis, simultaneous magnesium deficiency has the potential to intensify the seriousness of hypokalemia^26^. This complication is especially concerning given these patients' existing susceptibility stemming from dietary imbalances and insufficient intake of protein and energy^27^.

A positive linear correlation between Mg and K implied that both markers shared similar pathways associated with the changes in their concentrations. Because of the close interrelationship between Mg and K, we combined two markers, i.e., Mg × K, to amplify their association with long-term outcomes in chronic HD patients. We hypothesized that lower Mg and K in chronic HD patients share a similar pathway of high inflammation superimposed with malnutrition, which is linked to worse clinical outcomes.

Patients with lower Mg × K product were characterized by a higher prevalence of diabetes mellitus (DM), shorter HD vintage, lower nPCR, and a higher incidence of comorbid conditions. They also had lower serum albumin and prealbumin levels, and lower nPCR. These features indicated that HD patients with lower Mg × K are under a more profound malnutrition state with inadequate protein and calorie intake, which leads to a decrease in both Mg and K. In addition to higher hs-CRP levels, group 1 also had more comorbidities. This may be associated with the severity of hypoalbuminemia. In conclusion, we hypothesized that HD patients with lower Mg × K are in a more severe state of malnutrition-inflammation. In the multivariate analysis shown in Table 2, besides lower nPCR and higher hs-CRP, group 1 patients had lower serum creatinine levels, implying that patients with lower Mg × K might also tend to have muscle wasting. Therefore, we propose that chronic HD patients with lower Mg × K have diminished functional capacity related to metabolic stress, fulfilling the diagnostic criteria of protein energy wasting (PEW)^28^. The cutoff points of Mg (2.1 mg/dL vs. 2.4 mg/dL) and K (3.5 mmol/l vs. 4.0 mmol/l) between groups 1 and 2 are just a watershed of “subnormal” and “normal”, separating all the other 4 groups to the normal side in both markers. These four groups, i.e., groups 2,3,4 and 5 shared a similarity in nutritional, inflammatory status, and thus the long-term outcomes. This may also explain why group 1 showed a highest mortality rate.

The ISRNM expert has defined PEW to describe a “state of decreased body stores of protein and energy fuels”^28^. This abnormality is often associated with a diminished functional capacity related to metabolic stress. In this study, we found that patients with lower Mg × K were characterized by malnutrition, inflammation, and more comorbidities, leading to a more severe PEW state comparing to their higher Mg × K counterparts. This can explain the inferior outcomes in the groups with lower Mg × K. Group 1, with lower Mg × K, had a lower survival rate than the other four groups. Although a U-shaped relationship between serum K levels and death was reported in prevalent HD patients^28^, we found that the highest Mg × K, i.e., group 5 with higher serum albumin and prealbumin levels and lower hs-CRP, was not associated with a mortality risk.

Our study has several strengths. First, it is the first study to highlight the association between the Mg × K product and clinical outcomes in chronic HD patients. Second, all patients were followed for 2 years to monitor the constancy of Mg × K, confirming that it is an appropriate marker to evaluate prognosis of HD patients. However, our study also has several limitations that should be acknowledged. First, we could not distinguish between ionized, albumin-binding, and complex magnesium and we were unable to calculate the exact magnesium removal for each patient in this analysis. Second, 5% of patients were withdrawn during the study period due to incomplete data; this may be a confounding factor to our conclusions. Third, this study did not document the patients' residual kidney function. However, with an average dialysis duration more than 4.7 years and a decline rate of residual kidney function in hemodialysis patients of about 6 to 7% per month^29^, the included population is considered to have virtually no residual kidney function. Fourth, the small sample size in our study calls for careful interpretation of the results. It offers initial insights but may not fully reflect the intricate factors affecting mortality in hemodialysis patients, potentially limiting the generalizability of our findings. Fifth, a notable consistency in our study was the uniform use of Vitamin D3 across all five groups, indicating no significant variation. However, one limitation is the lack of data on the use of Calcium sensing receptor agonists, given that it's an out-of-pocket medication in Taiwan. Lastly, data on arterial blood gas, which may affect the level of serum [K], were lacking. However, we hypothesized that the level of acidemia in all patients might be similar, since the data were collected from stable ESRD patients before a routine HD session.

In conclusion, using the Mg × K product we demonstrate a useful strategy for risk stratification of HD patients. A parallel correlation between Mg and K was observed in this group of patients, and those with lower Mg tended to have lower K levels. Clinically stable dialysis patients with lower Mg × K product were characterized by shorter dialysis vintage, lower nPCR, hypoalbuminemia, and hypoprealbuminemia, and by higher hs-CRP levels, indicating that they were under a more profound malnutrition and inflammation status. After a follow-up of 2 years, patients with a lower Mg × K product were found to be associated with a higher mortality rate. We hypothesized that the Mg × K product may serve as a good surrogate biomarker to follow ESRD patients on maintenance HD.

## Methods

**Figure 4:**
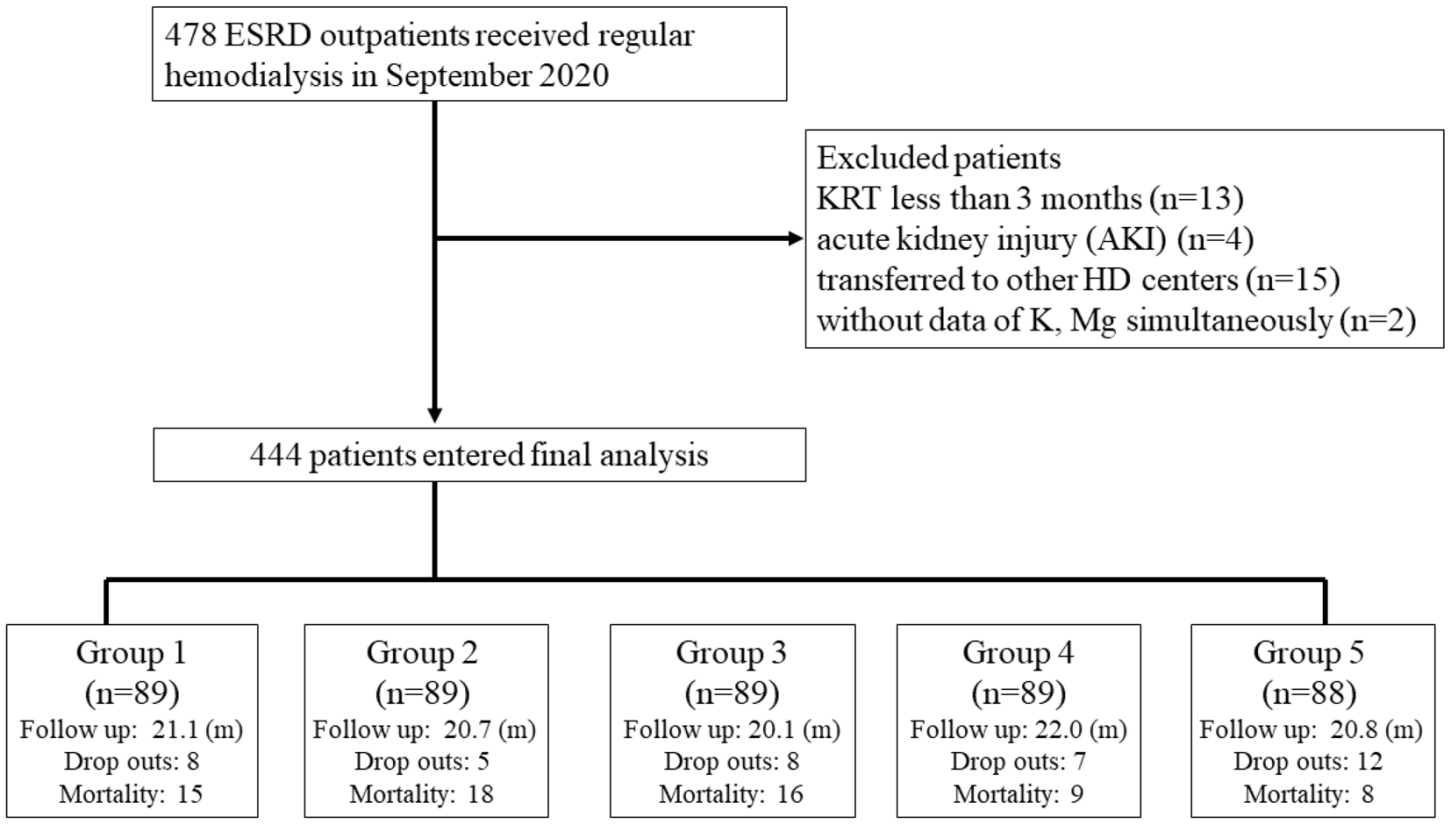
Flowchart of patient selection and group allocation based on Mg x K Product. ESRD outpatients receiving regular in-center HD, three times a week, 4 h a per session, at the Chi-Mei Medical Center, were screened using inclusion and exclusion criteria. Totally 444 patients were enrolled for final analysis. Individuals were divided into 5 groups according to Mg × K production. *Abbreviations* ESRD, End stage renal disease; HD, hemodialysis; KRT, Kidney replacement therapy; m, months.

This was a cohort observational study, collecting data prospectively. A total of 478 ESRD outpatients received uniform regimen of HD three times a week (4 h per session) at the Chi-Mei Medical Center in southern Taiwan. We excluded patients who had kidney replacement therapy for less than 3 months (n = 13), those with suspected acute kidney injury (n = 4), those transferred to other HD centers (n = 15), and those who did not have complete data for K and Mg (n = 2) (Fig. 4). We started to assess pre-HD serum K and Mg from September 2020, and following that consecutively every 3 months till June 2021. The total observation period was from September 5, 2020, to August 31, 2022. (Supplement Fig. 4) Data on serum K and Mg obtained during hospitalization or when patients visited the emergency department were excluded. Blood samples were obtained in the last mid-week of September 2020 at the initiation of the study, and following that every 3 months in subsequent 9 months. Total 4 sets of K and Mg were collected. Pre-HD biochemical analysis included serum urea nitrogen measured using the urease method, and creatinine (Cr, Kinetic Alkaline Picrate), sodium(Na), K (ion-selective electrodes), Mg (enzymatic method), cholesterol (enzymatic method), calcium (Ca, Arsenazo-III), phosphate (P, Phosphomolybdate), uric acid (UA, uricase), albumin (bromcresol green), intact parathyroid hormone (iPTH, Chemiluminescent Microparticle ImmunoAssay), hs-CRP (Turbidimetric method), and hemoglobin (Hb, sodium lauryl sulfate hemoglobin detection method).

Patients were divided into five groups according to their 1st serum Mg × K [Mg × K (t0)] at the last mid-week before HD in September 2020. The patients were categorized into group 1, bottom quintile, median Mg × K: 7.87 (n = 89); group 2, median Mg × K: 9.37 (n = 89); group 3, median Mg × K: 10.95 (n = 89); group 4, median Mg × K: 12.30 (n = 89); and group 5, top quintile, median Mg × K: 14.92(n = 88). The patients were followed up for a maximum of 2 years. The hollow-fiber dialyzers applied to all patients included REXEED-21A, REXEED-25A (Asahi Polysulfone, Japan), Polyflux 17 L, Polyflux 21 L (Gambro, Sweden), and FX 60 C, FX 80 C, FX 100 C (Fresenius, Germany). The formula of the dialysate bath was sodium, 139.0 mmol/L; calcium, 3.0 mmol/L; potassium, 2.0 mmol/L; magnesium,1.0 mmol/L; chloride, 106.5 mmol/L; acetate, 4.0 mmol/L; dextrose, 200 mg/dL, and bicarbonate, 39 mmol/L (Hemodialysis Concentrate A-35 & BP-11, Chi Sheng Chemical Corporation, Hsinchu, Taiwan). We also checked the nPCR of all participants. The comorbidity factors were recorded using the Davies comorbidity scores, and included coronary artery disease, congestive heart failure, peripheral vascular disease, stroke, neoplasm, chronic lung disease, liver cirrhosis, and hepatoma. These comorbidity factors were documented in the past medical records^30^.

### Statistical analysis

Continuous variables were reported as mean ± SD for normally distributed data, or as median (interquartile range) for non-normally distributed data. Baseline characteristics compared among the groups were analyzed using χ^2^ and ANOVA for categorical and continuous variables, respectively (Table 1). Compared to the other four groups as a whole, a stepwise forward multivariate logistic regression test was applied to evaluate the characteristics of group 1 and its association with various markers associated with nutritional and inflammation status, including HD vintage, serum albumin, serum phosphate, pre-HD Cr, nPCR, DM, sex, and age at the start of the study (Table 2). Cox proportional hazards models examined the factors associated with the product of Mg × K (Supplement Table 1) and evaluated the risk factors for predicting mortality (Table 3). In Table 3, model 1 included factors such as the Mg, K product, age, presence of diabetes mellitus (yes or no), sex (with male coded as 1), duration of hemodialysis treatment (hemodialysis vintage). Model 2 was constructed similarly to Model 1 but additionally incorporated the "davies comorbidity" factor. Model 3, building upon Model 2, further included "serum albumin" and "CRP" as covariates to robustly ascertain the potential predictors of mortality. Survival rates of the five groups categorized by the Mg × K product were analyzed using the Kaplan–Meier method, and log-rank tests were employed to compare the different survival curves between groups (Fig. 1). A two-tailed *p* value of less than 0.05 was considered to be significant. Computations were performed with the SPSS 22.0 package for Windows (IBM® SPSS® software, Chicago, IL, USA).

All individuals in this study were approved by the Chi-Mei Medical Center Institutional Review Board (10911-004), and the study was conducted according to the Declaration of Helsinki. Written informed consent was obtained from all participants.

### Supplementary Information

Supplementary Information.

## Data Availability

All data generated or analyzed during this study are included in this published article.
