# Supplementary material for: Lower product of magnesium × potassium is associated with higher mortality in chronic hemodialysis patients: a cohort study
Source: Sci Rep. 2023 Dec 13;13:22128. doi: 10.1038/s41598-023-49372-y (PMC10719325; doi:10.1038/s41598-023-49372-y)
Supplement: Supplementary file 1 — Supplementary Information. [file 41598_2023_49372_MOESM1_ESM.docx]

**Supplement to “Lower Product of Magnesium x Potassium is Associated with Higher Mortality in Chronic Hemodialysis Patients: A Cohort Study”**

Authors: Jui-Yi Chen, Ming-Yan Jiang, Yun-Ting Huang, Jyh-Chang Hwang

**Supplement Table 1**. Factors association with the Product of Potassium x Magnesium

|  | Univariate | |  |  |  | Multivariate | |  |  |  |
| --- | --- | --- | --- | --- | --- | --- | --- | --- | --- | --- |
|  |  | 95% CI |  | *p* |  |  | 95% CI |  | *p* |  |
|  | Beta | lower | upper |  |  | Beta | lower | upper |  |  |
| Age, year | -0.035 | -0.031 | 0.014 | 0.461 |  | -0.049 | -0.035 | 0.011 | 0.315 |  |
| Sex, male=1 | -0.077 | -1.013 | 0.097 | 0.106 |  | -0.062 | -0.941 | 0.209 | 0.212 |  |
| HD vintage, year | 0.079 | -0.007 | 0.089 | 0.096 |  | 0.046 | -0.026 | 0.074 | 0.346 |  |
| nPCR, g/kg/day | 0.204 | 1.106 | 3.05 | <0.001 |  | 0.206 | 1.123 | 3.064 | <0.001 |  |
| hs-CRP, mg/L | -0.152 | -0.032 | -0.008 | 0.001 |  | -0.122 | -0.035 | -0.004 | 0.012 |  |
| Albumin, g/dL | 0.284 | 1.568 | 3.018 | <0.001 |  |  |  |  |  |  |
| Prealbumin, mg/dL | 0.195 | 0.039 | 0.11 | <0.001 |  |  |  |  |  |  |
| BUN, mg/dL | 0.291 | 0.034 | 0.064 | <0.001 |  |  |  |  |  |  |
| Creatinine, mg/dL | 0.206 | 0.117 | 0.305 | <0.001 |  |  |  |  |  |  |
| Phosphate, mg/dL | 0.307 | 0.472 | 0.857 | <0.001 |  |  |  |  |  |  |
| Abbreviations: HD: hemodialysis, nPCR: normalized protein catabolism rate, hs-CRP: high sensitivity-C reactive protein, BUN: blood urea nitrogen | | | | | | | | | | |

**Supplement Table 2**: Review of Literature on the Importance of Magnesium (Mg) and Potassium (K) in Hemodialysis Patients

| Condition | Consequences in Hemodialysis Patients | Underlying Issues | Association with Mortality | References |
| --- | --- | --- | --- | --- |
| Hypomagnesemia | - Endothelial dysfunction  - Vascular calcification  Atherosclerosis  - Insulin resistance  - Metabolic syndrome | - Malnutrition  - Inflammation | Increased risk of cardiovascular disease and impaired regulation of insulin-mediated glucose uptake and vascular tone modulation, leading to higher mortality rates. | ^1^  ^2^ |
| Hypokalemia | - Protein-energy wasting (PEW) | - Lack of nourishment  - Various degrees of inflammation | Associated with conditions like PEW that significantly contribute to increased mortality. | ^3^ |

**Supplement Figure 1.** The study protocol followed a clear, sequential timeline


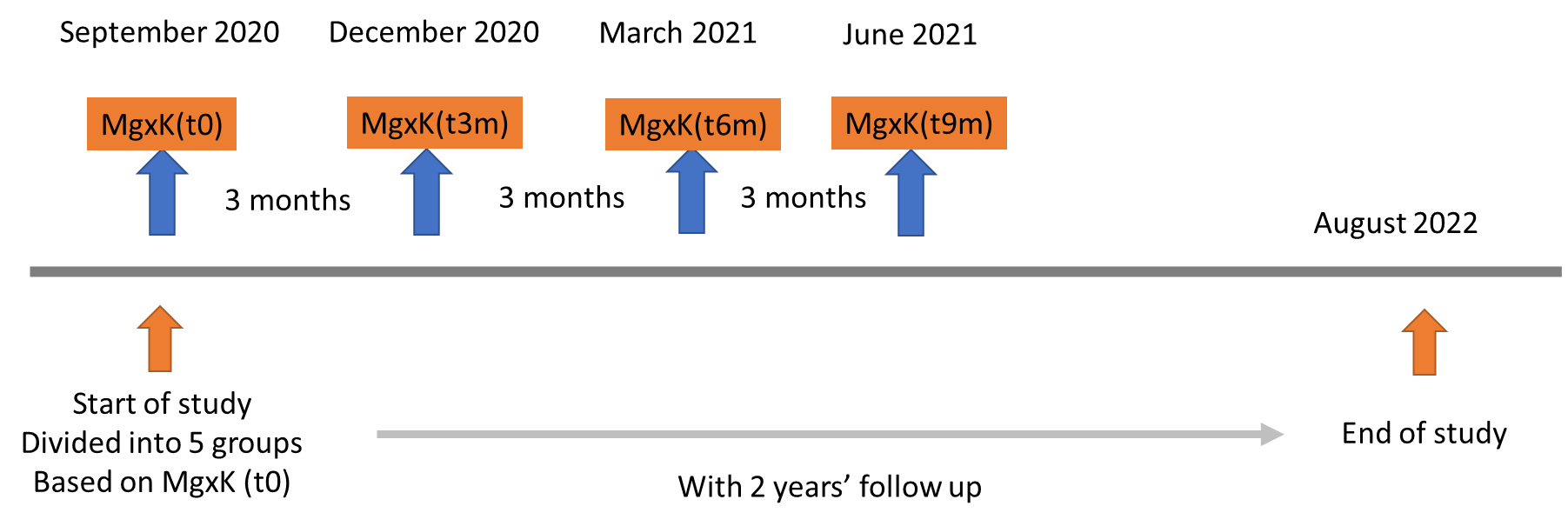


**References**

1 Hashimoto, T. *et al.* Serum magnesium, ambulatory blood pressure, and carotid artery alteration: the Ohasama study. *Am J Hypertens* **23**, 1292-1298, doi:10.1038/ajh.2010.168 (2010).

2 Maraj, M. *et al.* Malnutrition, Inflammation, Atherosclerosis Syndrome (MIA) and Diet Recommendations among End-Stage Renal Disease Patients Treated with Maintenance Hemodialysis. *Nutrients* **10**, doi:10.3390/nu10010069 (2018).

3 Fouque, D. *et al.* A proposed nomenclature and diagnostic criteria for protein-energy wasting in acute and chronic kidney disease. *Kidney Int* **73**, 391-398, doi:10.1038/sj.ki.5002585 (2008).
